# Therapeutic hypothermia in cerebral air embolism: a case report

**DOI:** 10.1186/2193-1801-2-411

**Published:** 2013-08-28

**Authors:** Jochen Bäuerle, Andreas Fischer, Tobias Hornig, Karl Egger, Tobias Wengenmayer, Jürgen Bardutzky

**Affiliations:** Department of Neurology, University Medical Center Freiburg, Breisacher Str. 64, Freiburg, 79106 Germany; Department of General and Visceral Surgery, University Hospital Freiburg, Freiburg, Germany; Department of Neuroradiology, University Hospital Freiburg, Freiburg, Germany; Department of Cardiology and Angiology, University Hospital Freiburg, Freiburg, Germany

**Keywords:** Cerebral air embolism, Therapeutic hypothermia, CT, MRI

## Abstract

**Background:**

Cerebral air embolism is a life-threatening complication of various diagnostic and therapeutic procedures. Hyperbaric oxygenation is considered to be the cornerstone of its treatment.

**Case description:**

We report a patient with cerebral air embolism after endoscopy of a perineal abscess. Immediate CT imaging confirmed the diagnosis and MRI showed cortically localized areas of restricted diffusion along the gyri. Since hyperbaric oxygenation was not available, moderate hypothermia was applied for neuroprotection.

**Conclusion:**

This case illustrates a rare complication of endoscopic interventions, and imaging characteristics of cerebral air embolism were described. Furthermore, we discuss the potential utility of therapeutic hypothermia in cerebral air embolism.

## Background

Cerebral air embolism (CAE) is a life-threatening complication of various diagnostic and therapeutic procedures. Diagnosis is difficult to establish and depends primarily on clinical experience. We report a patient with CAE after endoscopic bouginage of a perineal abscess treated with hypothermia immediately after diagnosis.

## Case report

A 59-year-old man developed sudden respiratory distress, tachycardia, hypotonia, and coma about one minute after flexible endoscopy with dilatation and irrigation of a perineal abscess under sedation with propofol. Two years ago he had undergone pelvic exenteration because of advanced prostate cancer with intraoperative (15 Gy) and adjuvant radiotherapy (63 Gy). Wound healing was complicated by a perineal wound infection and development of a perineal abscess. Afterwards, periodical bouginage by flexible endoscopy (Hegar bouginage) of the abscess fistula had been necessary in order to drain pus and to keep the fistula open.

**Figure 1:**
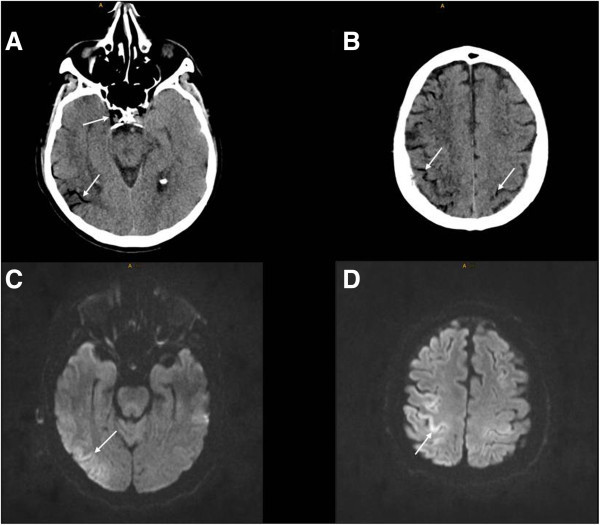
**CT and MRI in cerebral air embolism.** The initial head CT shows air entrapment in cortical branches of both middle cerebral arteries and in the cavernous sinus (arrows in **A** and **B**). On day ten MRI of the brain demonstrates cortically localized areas of restricted diffusion along the gyri **(**arrows in **C** and **D)**.

After a few seconds tachycardia ceased spontaneously but neurologic examination revealed a comatose patient with ping-pong gaze, decerebrate rigidity and positive Babinski response bilaterally. Mechanical ventilation was initiated with 100% oxygen since vascular air embolism was suspected. Furthermore, the patient was placed in partial left lateral decubitus position in order to entrap possible air in the right ventricle. Head CT showed massive air entrapment in cortical branches of both middle cerebral arteries, predominantly on the right side, and in the cavernous sinus (Figure 1). Since the patient was in need of critical care medicine we were not able to administer hyperbaric therapy. Thus, in order to reduce brain injury we decided to apply moderate hypothermia with a target bladder temperature of 33°C for 24 hours. Hypothermia was immediately induced with rapid free floating ice cold saline infusions (4°C, 25 ml/kg body weight) and after the diagnostic procedures by an external cooling system. Transthoracic echocardiography detected no intraventricular air. One hour and fifteen minutes after the first imaging, CT angiography showed normal cerebral blood vessels and complete resolution of the intracranial air. During rewarming the patient developed generalized myoclonus which did not respond to intravenous valproic acid, phenytoin and levetiracetam. Only propofol resulted in a discontinuation of myoclonus. The following days the patient increasingly regained consciousness and could be extubated on day ten. At that time MRI of the brain demonstrated multiple bihemispheric and cortially localized areas of restricted diffusion along the gyri (Figure 1). Sixteen days after the event the patient was discharged to a rehabilitation facility with a multimodal neglect and a moderate hemiparesis of the left extremities. Three months later his Rankin scale score remained 4.

## Discussion and conclusion

This case illustrates that systemic air embolism should be considered as cause of neurological and cardiopulmonary deterioration during pelvic interventions (Mirski et al. 2007).

In our patient we assume that insufflated air entered the venous circulation via a disruption of the endothelial surface of the abscess and passed perineal venous vessels which drain into the inferior vena cava. The initial head CT of our patient showed air in both the arterial and the venous system. Arterial air entrapment is suggestive for a patent foramen ovale as cause for paradoxical air embolism. Additionally, intrapulmonary shunts and transcapillary passage of air have been proposed as alternative mechanisms, especially in cases with large venous air volumes (Mirski et al. 2007). We did not perform a transesophageal echocardiography for detection of an intra cardiac shunt, since this would not have implied any therapeutic consequences for our patient. Furthermore, air accumulation in the cavernous sinus may be explained by jugular valve insufficiency and retrograde air passage through the internal jugular veins (Nedelmann et al. 2007).

Our report emphasizes that unenhanced CT of the head is capable to detect CAE if performed directly after symptom onset. Immediate brain imaging is thus required to verify the diagnosis. However, the absence of intracerebral air on CT does not exclude the diagnosis (Muth and Shank 2000). MRI demonstrated cortical ischemia predominately in the territory of the right middle cerebral artery. The gyriform pattern of restricted diffusion has been observed in several cases of CAE previously (Caulfield et al. 2006; Verro 2010; Koster et al. 2012) and may be the result of end-arterial occlusion and endothelial damage by air bubbles (Sobolewski et al. 2012). Interestingly, these cortical DWI changes resemble laminar necrosis on MRI in patients with hypoxic brain injury. If this finding exhibits a diagnostic value of CAE in cases without detection of intracerebral air on CT remains unclear and should be evaluated in prospective studies.

At present, hyperbaric oxygenation is considered to be the cornerstone of CAE treatment (Muth and Shank 2000; Mirski et al. 2007). However, prospective trails demonstrating its efficacy are lacking. In our case hyperbaric oxygenation was not available. Since induced hypothermia after cardiac arrest provides substantial neuroprotective effects (Hypothermia after Cardiac Arrest Study Group 2002), and CAE may induce pathophysiological changes similar to hypoxic brain injury, we decided to apply moderate hypothermia for neuroprotection. We are aware of the limited significance of this case report since we are not able to estimate the effect of hypothermia on the course of our patient. Otherwise, the initial neurologic status heralded a poor outcome of our patient and he retained a moderately severe disability after 3 months. Similarly, Inoue et al. reported a favorable outcome of a patient with CAE after pleural lavage treated with therapeutic hypothermia (Inoue et al. 2013). Therefore, we conclude that hypothermia is feasible in CAE and may have a neuroprotective effect in this disorder. In contrast to hyperbaric oxygenation, permanent availability makes hypothermia highly beneficial. Thus, as potential neuroprotective approach in CAE therapeutic hypothermia should be further investigated.

## Consent

Written informed consent was obtained from the patient for publication of this case report.
